# Influence of job satisfaction on SRH and happiness among Korean disabled workers: findings from the panel survey of employment for the disabled 2016–2018

**DOI:** 10.3389/fpubh.2023.1122648

**Published:** 2023-09-15

**Authors:** Young Lim Lee, Jeong Min Yang, Jae-Hyun Kim

**Affiliations:** ^1^Department of Psychology and Psychotherapy, Dankook University, Cheonan, Republic of Korea; ^2^Institute for Digital Life Convergence, Dankook University, Cheonan, Republic of Korea; ^3^Department of Public Health, General Graduate School of Dankook University, Cheonan, Republic of Korea; ^4^Department of Health Administration, College of Health Science, Dankook University, Cheonan, Republic of Korea

**Keywords:** job satisfaction, SRH, happiness, persons with disability, South Korea

## Abstract

**Background:**

An understanding of the economic life activities of persons with disabilities (PWD) is important. Their ability to perform tasks and an increase in their income are more likely to yield an improvement in their Self-Rated Health (SRH) and happiness. However, there is still a lack of understanding of the specific associations among PWD in South Korea. Thus, this study conducted a longitudinal analysis of the association between job satisfaction and SRH, happiness among the Korean PWD.

**Methods:**

After excluding missing values, data on 1,637 participants at baseline (1st wave) were analyzed using the chi-square test, t-test, Analysis of Variance (ANOVA) and generalized estimating equation (GEE) model for data from 1st to 3rd Panel Survey of Employment for the Disabled (PSED). All analyses were conducted using the SAS statistical software package, version 9.4.

**Results:**

Compared to very high job satisfaction group, low job satisfaction group was more likely to experience negative SRH [odds ratio (OR): 3.497, value of *p*: <0.0001] and experience low happiness (*B*: −0.291, value of *p*: <0.0001). Furthermore, in terms of the overall satisfaction with current job among the PWD, compared to the ‘very satisfied’ group, ‘very unsatisfied’ group had higher negative SRH (OR: 5.158, value of *p*: 0.003) and lower happiness (*B*: −0.327, value of *p*: <0.0001).

**Conclusion:**

This study suggests that increasing job satisfaction of PWD possibly leads to decreased negative SRH and to increased happiness, resulting in better SRH and happiness. Furthermore, it suggests the establishment of systemic, policy-oriented measures to enhance the employment opportunities for disabled individuals in Korea and create an inclusive working environment that aligns with their respective job responsibilities.

## Background

Recently, people have increasingly begun to realize that they can enjoy a high-quality life, by maintaining a healthy lifestyle in which their physical health, happiness, and social stability are balanced. One of the major activities of modern industrialized societies is employment, as this is an essential component in ensuring equal opportunities in socioeconomic life (1). Especially, not only in economic activities, but job satisfaction is one of the crucial factors for people with disabilities (PWD). According to previous research (2, 3), the management of job satisfaction among PWD is essential due to complaints arising from organizational structural aspects and income distribution. Also, Unemployment is bound to be characterized by poor psychological and physical health and other problems (4–7). Since income is one of the most significant factors to maintain socioeconomic status, lower incomes might lead to distress arising from social and psychological deprivation, resulting in poor health (5).

The Convention on the Rights of Persons with Disabilities (CRPD) (8) and the enactment of laws to guarantee human rights, including the Disability Discrimination Act (DDA) (9), were intended to raise awareness of disabilities and the human right of the persons with disabilities (PWD) and to strive for social integration.

It is widely assumed that higher employment of PWD improves their subjective well-being. An understanding of the economic life activities of PWD is important. Demonstrations of their ability to perform particular tasks and an increase in their income are more likely to yield an improvement in their quality of life (10). A panel study conducted by Choi, Kim, Han, and Kim (2019) (11) suggests that the lower income of PWD in South Korea and the precarious nature of their employment are significantly related to poor SRH compared with those who have more stable employment or higher incomes. Van Campen and Iedema (12), however, found that employment of PWD was much less closely associated with perceived health and happiness than expected. In fact, they argued that objective aspects of work, such as the number of hours they work in paid employment, have little effect on perceived health and well-being compared to subjective aspects of work, such as the extent to which they enjoy their work. Thus, the job satisfaction of PWD might be a more important factor in promoting better well-being and perceived health than simply the income associated with their work.

Well-being is often regarded as a term that is similar in meaning to happiness (13). According to the recent World Happiness Report released by the United Nations (UN) (14), South Korea ranked 57th out of 137 countries in terms of happiness index, placing it among the lowest-ranking OECD member countries. In particular, by scoring low on social support and generosity, among the six indicators of the World Happiness Index, South Korea falls on the lower end among all countries, indicating a relatively low level within the social and cultural context (14). Furthermore, according to previous research in Korea, comparing the happiness trajectories between PWD and those non-PWD, it has been reported that the happiness index of the disabled group is lower than that of the non-disabled group also, Various factors influencing happiness, such as SRH status, self-esteem, and satisfaction with social relationships, were found to be deteriorated in the disabled group (15).

Happiness is a multidimensional construct that includes cognitive assessment (such as satisfaction with one’s life) and affective assessment (such as moods and emotions) (16, 17). The pursuit of happiness is one of the most important goals for many people, and happiness is closely related to health (18). It has been claimed that SRH is more reliable than objective health status in a person’s self-evaluation of their own physical and psychosocial status (19). The finding that well-being was strongly associated with SRH (20), but not with objective health (21) suggests that SRH is more important to well-being than objective health. Park et al. (22) investigated whether job satisfaction and security are associated with SRH and well-being of Korean employees. They found that Korean workers who are more satisfied with and secure in their jobs demonstrated good SRH and well-being, even when they are exposed to ergonomic and psychosocial hazards at work which negatively affect their SRH and well-being. A study that evaluated Portuguese workers also showed similar results in which job satisfaction is related to health, happiness, and subjective well-being (23). In addition, the researchers found that job satisfaction has a protective effect on health, happiness, and subjective well-being. This implies that higher job satisfaction potentially can improve workers’ health, happiness, and subjective well-being.

Although job satisfaction has been evaluated in relation to SRH, and happiness or well-being for non-PWD, there have been a few studies focused on PWD in the society. in Korean society, research has mainly focused on the factors influencing job satisfaction among PWD (24–26), and as far as we know, there have been no studies conducted on the impact of job satisfaction on SRH. Therefore, to better understand the experiences of PWD and improve their quality of life, it is necessary to examine how job-related factors, such as job satisfaction, are associated with those related to daily life satisfaction, such as SRH and happiness.

By referring to the prior references and other study framework (24–26), we have selected control variables for analyzing the relationship among PWD. In this study, we hypothesize that lower job satisfaction among PWD in South Korea will be associated with lower SRH and happiness. To investigate this, we aim to analyze the association between job satisfaction and SRH or happiness among PWD using the 1st–3rd waves of the Panel Study of Employment for the Disabled (PSED). Through this analysis, the goal of this research is to present these findings as fundamental data for policy and institutional measures aimed at preventing the exacerbation of mental health issues within this specific subgroup of PWD.

## Methods

### Study sample and design

Data were obtained from 1st–3rd data (2016–2018 year) for 2nd wave Panel Survey of Employment for the Disabled (PSED) (27). The PSED is the first nationally representative longitudinal survey of PWD in South Korea, and nationwide data was collected using a computer-assisted personal interviewing program (CAPI). As of May 15, 2016, about 4,577 registered disabled persons stipulated in Article 2 of the Welfare Act for PWD aged 15 to 64 years old residing nationwide were targeted. In the first baseline survey in 2016, 4,577 individuals from 2,056 households (44.9 per household) were interviewed. The second survey, in 2017, followed up with 4,214 participants, who represented 92.1% of the original panel. The third survey, in 2018, followed up with 4,104 participants, who represented 89.6% of the original panel.

**Figure 1:**
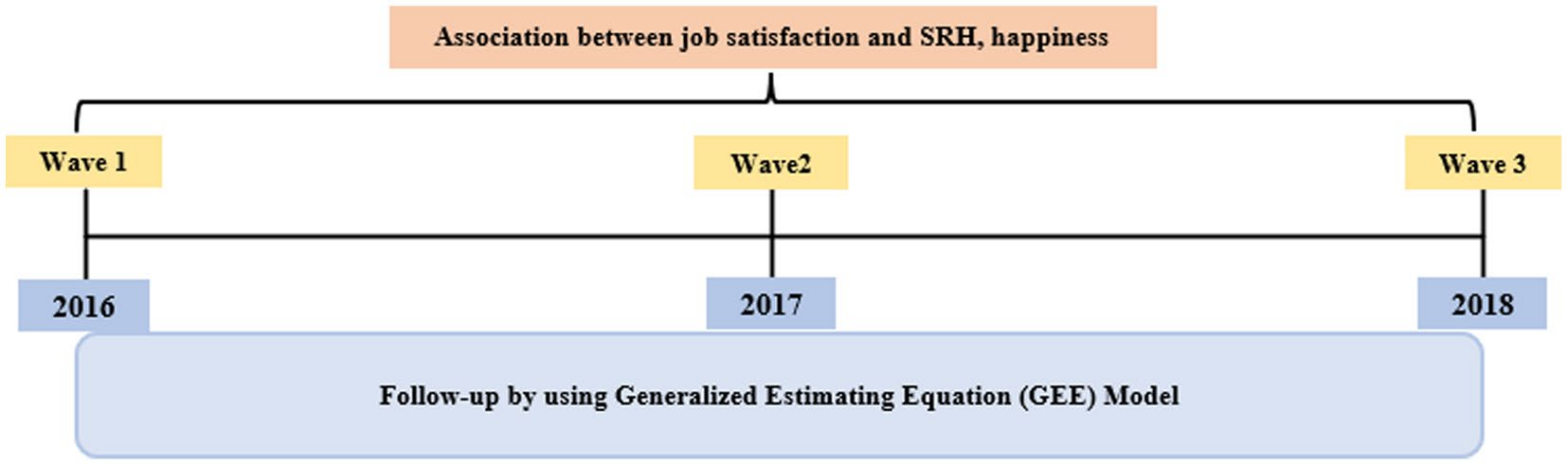
The study timeline.

The survey includes demographic data (gender, age, education, disability status, disability grade, disability type) and data regarding economic participation. In addition, the individual and environmental factors that influence economic activity are included. For the current study, 1st–3rd data (2016–2018 year) for 2nd wave Panel Survey data were used, as this data includes all measures and variables aligned with the research questions and hypotheses being tested. To estimate the association between job satisfaction and SRH and happiness among disabled who are engaged in economic activities, we included 1,637 participants with no missing information at baseline (the 2016 PSED). Figure 1 displays the timeline and procedure of this study.

### Independent variables

### Job satisfaction

Job satisfaction, our independent variable of interest, was measured in 10 variables. Briefly (1) wage or income (1, very dissatisfied, 2, dissatisfied, 3, moderate, 4, satisfied, 5: very satisfied), (2) Job security, (3) what you are doing (4) working environment (5) working hours (6) own development potential (7) communication (8) fairness of personnel evaluation (9) welfare benefits (10) convenience for disability. Thus, they are summed and the resulting total ranging from 10 to 50 used. Finally, job satisfaction was grouped into four groups: <20: low job satisfaction, 20–29: medium job satisfaction, 30–39: high job satisfaction and > 39: very high job satisfaction. The validity and reliability of the measurement for that variable were found to be appropriated (28). Specifically, the Tucker-Lewis Index (TLI) and Comparative Fit Index (CFI) coefficients are used as criteria to assess the goodness of fit of a model. If these coefficients are above 0.9, the model is considered to be good. The Root Mean Square Error Approximation (RMSEA) below 0.9 indicates a good fit. In this analysis, the model fit for Job Satisfaction was determined to be good with TLI: 0.966, CFI: 0.938, and RMSEA: 0.80.

### Overall satisfaction with the current job

Overall satisfaction was measured in only one question. The responses were assigned to 1 of 5 subcategories: 1: very dissatisfied, 2: dissatisfied, 3: moderate, 4: satisfied, 5: very satisfied. According to the reliability analysis conducted in this study for overall satisfaction with the current job, the Cronbach’s alpha coefficient was found to be 0.925.

### Dependent variables

### Self-rated health

Perceived physical health status was assessed with the question: “How do you usually perceive your health?” The response “insufficient” or “very insufficient” indicated “Bad,” and the response “normal,” “sufficient,” or “very sufficient” indicated “Good,” thus dichotomizing the response for logistic regression analysis (29).

### Level of happiness in mental health

Level of happiness in mental health measures a continuum of subjective probabilities. The response to the question ranges from 0 to 10, where 0 means you are very unhappy, and 10 meaning you are very unhappy, and it is divided into 10 points.

### Control variables

Gender was divided into male and female and age groups were divided into three categories: 15–29, 30–39, 40–49, 50–59, and ≥ 60 years. Residential region was categorized into metropolitan (Seoul), urban (Daejeon, Daegu, Busan, Incheon, Kwangju, or Ulsan), and rural (otherwise). Marital status was divided into three groups: Single, Married and separation or separation. Smoking status (never, former smoker, and smoker), alcohol consumption (never, former drinker and drinker) was categorized three groups, respectively. Stress status was divided into 3 groups: very, less, low. In the case of disability level, it is divided into two groups: severe (level 1 to level 3) and light (level 4 to level 6) and disability type was also divided into two groups: physical disability and other based on the number of samples of the disabled.

### Analytical approach and statistics

Chi-square test, *t*-test, ANOVA and generalized estimating equation (GEE) model were used to investigate the association between job satisfaction and SRH and happiness. The differences between the characteristics of the respondents were examined using the chi-square test. value of *p* <0.05 was considered statistically significant. Participants who responded repeatedly three times were included in the study, and all variables (independent, dependent, and control variables) were measured three times. Therefore, a generalized estimating equation (GEE) model was used to examine the association between job satisfaction and happiness, SRH. The GEE model was used to analyze the variation within individuals of repeated measurement variables (30, 31). For the analysis using the GEE model, the SAS procedure “PROC GENMOD” was used, and the best model was selected by checking the working correlation structure (32, 33). Analyses with GEE were expressed as odds ratio (OR) and 95% confidence interval (CI). All statistical analyses were performed using SAS statistical software package version 9.4 (SAS Institute Inc., Cary, NC, United States).

## Results

### Prevalence of SRH and happiness

Table 1 displays the descriptive statistics of all variables at baseline (2016). Of the 1,637 research subjects included in our study, the prevalence of poor SRH was 28.3% (464 participants) (Table 1). Of the total sample, 48.2% (13 participants) of those with low job satisfaction (< 20) had poor SRH and 15.5% (43 participants) of those with very high job satisfaction (> 39) had poor SRH. Mean score of happiness was 6.471 (SD: 1.479). Of the total sample, mean score of happiness in mental health of those with low job satisfaction (< 20) had 5.370 (SD: 1.418) and mean score of happiness in mental health of those with very high job satisfaction (> 39) had 7.198 (SD: 1.373).

**Table 1:** General characteristics of subjects included for analysis at baseline.

	Total		Self-rated health						Happiness		
			Poor			Good		*p*-value			*p*-value
	*N*	%	*N*		%	*N*	%		Mean	SD	
Job satisfaction								<0.0001			<0.0001
<20	27	1.7	13		48.2	14	51.9		5.370	1.418	
20–29	411	25.1	179		43.6	232	56.5		5.788	1.518	
30–39	921	56.3	229		24.9	692	75.1		6.587	1.350	
>39	278	17.0	43		15.5	235	84.5		7.198	1.373	
Gender								0.0004			0.826
Male	1,240	75.8	324		26.1	916	73.9		6.452	1.484	
Female	397	24.3	140		35.3	257	64.7		6.526	1.464	
Age								<0.0001			0.282
15–29	241	14.7	49		20.3	192	79.67		6.564	1.645	
30–39	513	31.3	98		19.1	415	80.9		6.601	1.392	
40–49	529	32.3	160		30.3	369	69.8		6.474	1.445	
50–59	246	15.0	102		41.5	144	58.5		6.203	1.530	
>59	108	6.6	55		50.9	53	49.1		6.231	1.457	
Residential region								0.4497			0.468
Metropolitan	341	20.8	101		29.6	240	70.4		6.441	1.602	
Urban	416	25.4	125		30.1	291	70.0		6.505	1.395	
Rural	880	53.8	238		27.1	642	73.0		6.465	1.470	
Marital status								<0.0001			<0.0001
Married	908	55.5	242		26.7	666	73.4		6.678	1.403	
Single	546	33.4	139		25.5	407	74.5		6.347	1.523	
Divorce, separated	183	11.2	80		45.4	100	54.6		5.803	1.484	
Smoking status								0.4773			0.022
Current smoker	475	29.0	129		27.2	346	72.8		6.244	1.504	
Former smoker	367	22.4	113		30.8	254	69.2		6.572	1.508	
Nothing	795	48.6	222		27.9	573	72.1		6.559	1.438	
Alcohol consumption								<0.0001			0.857
Drinker	951	58.2	226		23.8	725	76.2		6.435	1.485	
Former drinker	258	15.8	102		39.5	156	60.5		6.527	1.471	
Nothing	426	26.1	135		31.7	291	68.3		6.514	1.473	
Stress status								<0.0001			<0.0001
Hardly feel	195	11.9	30		15.4	165	84.6		7.246	1.400	
Moderate	529	32.3	111		21.0	418	79.0		6.712	1.295	
Almost feel a lot	913	55.8	323		35.4	590	64.6		6.165	1.510	
Disability grade								0.1789			0.387
1–3	414	25.3	128		30.9	286	69.1		6.426	1.505	
4–6	1,223	74.7	336		27.5	887	72.5		6.485	1.471	
Disability type								0.5894			0.398
Physical disability	946	57.8	273		28.9	673	71.1		6.536	1.452	
Other	691	42.2	191		27.6	500	72.4		6.380	1.512	
Total	1,637	100.0	464		28.3	1,173	72		6.471	1.479	

*Hypertension, diabetes, cancer, chronic obstructive pulmonary disease, liver disease, cardiovascular disease, cerebrovascular disease, arthritis.

### Association between job satisfaction and SRH and happiness

Table 2 shows the results of the panel data analysis using GEE model, which investigated the association between job satisfaction and SRH, Happiness. After adjusting for all of these confounders, odds ratio of poor SRH of those with low job satisfaction (< 20) was 3.497 times [odds ratio (OR): 3.497, 95% Confidence Interval (CI): 1.960–6.239, value of *p* <0.0001], medium job satisfaction (20–29) was 2.671 times (OR: 2.671, 95% CI: 2.116–3.371, value of *p* <0.0001) and high job satisfaction (30–39) was 1.398 times (OR: 1.398, 95% CI: 1.131–1.727, value of *p*: 0.0002) higher compared with those with very high job satisfaction (> 39). In terms of happiness, estimate of those with low job satisfaction (< 20) was – 0.291 points lower (*B*: -0.291, 95% CI: −0.354,-0.227, value of *p*: <0.0001), medium job satisfaction (20–29) was −0.182 points lower (*B*: -0.182, 95% CI: −0.200,-0.164, value of *p*: <0.0001) and high job satisfaction (30–39) was −0.076 points lower (*B*: -0.076, 95% CI: −0.089,-0.062, value of *p*: <0.0001) compared with those very high job satisfaction (> 39) (Table 2).

**Table 2:** Effect of job satisfaction on self-rated health and happiness.

	Self-rated health				Happiness			
	*OR*	95% CI		*P*-value	*B*	95% CI		*P*-value
Job satisfaction								
<20	3.497	1.960	6.239	<0.0001	-0.291	−0.354	−0.227	<0.0001
20–29	2.671	2.116	3.371	<0.0001	−0.182	−0.200	−0.164	<0.0001
30–39	1.398	1.131	1.727	0.002	−0.076	−0.089	−0.062	<0.0001
>39	1.000				ref			
Gender								
Male	0.761	0.626	0.926	0.006	0.002	−0.014	0.017	0.820
Female	1.000				ref			
Age								
15–29	0.168	0.117	0.243	<0.0001	0.082	0.053	0.112	<0.0001
30–39	0.234	0.176	0.311	<0.0001	0.056	0.032	0.080	<0.0001
40–49	0.366	0.281	0.476	<0.0001	0.047	0.023	0.071	<0.0001
50–59	0.584	0.445	0.767	0.000	0.016	−0.009	0.041	0.210
>59	1.000				ref			
Residential region								
Metropolitan	0.915	0.760	1.102	0.349	−0.005	−0.019	0.010	0.518
Urban	1.074	0.909	1.268	0.401	0.010	−0.003	0.023	0.127
Rural	1.000				ref			
Marital status								
Married	0.531	0.430	0.654	<0.0001	0.099	0.080	0.119	<0.0001
Single	0.818	0.631	1.061	0.130	0.017	−0.006	0.041	0.144
Divorce, separated	1.000				ref			
Smoking status								
Current smoker	1.087	0.882	1.338	0.435	−0.010	−0.026	0.006	0.226
Former smoker	1.038	0.839	1.284	0.730	0.003	−0.013	0.019	0.718
Nothing	1.000				ref			
Alcohol consumption								
Drinker	0.686	0.565	0.834	0.000	−0.008	−0.024	0.007	0.294
Former drinker	1.115	0.891	1.396	0.341	−0.002	−0.020	0.017	0.861
Nothing	1.000				ref			
Stress status								
Hardly feel	0.303	0.228	0.403	<0.0001	0.129	0.112	0.147	<0.0001
Moderate	0.527	0.452	0.614	<0.0001	0.050	0.038	0.062	<0.0001
Almost feel a lot	1.000				ref			
Disability grade								
1–3	1.414	1.193	1.676	<0.0001	−0.006	−0.020	0.008	0.396
4–6	1.000				ref			
Disability type								
Physical disability	1.337	1.146	1.559	0.000	0.006	−0.007	0.018	0.370
Other	1.000				ref			
Year								
2016	1.196	1.012	1.415	0.036	−0.017	−0.031	−0.004	0.011
2017	0.929	0.781	1.105	0.404	0.003	−0.011	0.016	0.671
2018	1.000				ref			

### Association between overall job satisfaction and SRH and happiness

Table 3 adjusted for socioeconomic status and health status and risk behaviors variables. After adjusting for all of these confounders, odds ratio of poor SRH of those with “Very dissatisfied” was 5.158 times (OR: 5.158, 95% CI: 1.740–15.290, value of *p*: 0.003), “Unsatisfied” was 2.643 times (OR: 2.643, 95% CI: 1.510–4.626, value of *p*: 0.001) higher compared with those with “Very satisfied.” In terms of happiness in mental health, estimate of those with “Very dissatisfied” was – 0.327 lower (*B*: -0.327, 95% CI: −0.437,-0.217, value of *p* <0.0001), “Unsatisfied” was – 0.266 lower (*B*: -0.266, 95% CI: −0.307,-0.225, value of *p* <0.0001) compared with “Very satisfied” (Table 3).

**Table 3:** Effect of Overall satisfaction on self-rated health and happiness.

	Self-rated health					Happiness			
	*OR*	95% CI		*P*-value		*B*	95% CI		*P*-value
Overall satisfaction with current job									
Very unsatisfied	5.158	1.740	15.290	0.003		−0.327	−0.437	−0.217	<0.0001
Unsatisfied	2.643	1.510	4.626	0.001		−0.266	−0.307	−0.225	<0.0001
Usually	1.406	0.846	2.340	0.189		−0.161	−0.193	−0.128	<0.0001
Satisfied	0.817	0.490	1.360	0.437		−0.058	−0.089	−0.026	0.000
Very satisfied	1.000					ref			
Gender									
Male	0.774	0.636	0.942	0.011		0.001	−0.015	0.016	0.934
Female	1.000					ref			
Age									
15–29	0.159	0.110	0.229	<0.0001		0.085	0.056	0.115	<0.0001
30–39	0.221	0.166	0.294	<0.0001		0.063	0.039	0.087	<0.0001
40–49	0.354	0.272	0.460	<0.0001		0.051	0.027	0.074	<0.0001
50–59	0.570	0.434	0.749	<0.0001		0.020	−0.005	0.045	0.112
>59	1.000					ref			
Residential region									
Metropolitan	0.917	0.762	1.104	0.362		−0.004	−0.018	0.010	0.588
Urban	1.095	0.927	1.294	0.284		0.009	−0.005	0.022	0.198
Rural	1.000					ref			
Marital status									
Married	0.522	0.424	0.644	<0.0001		0.102	0.083	0.122	<0.0001
Single	0.812	0.626	1.053	0.116		0.023	0.000	0.046	0.054
Divorce, separated	1.000					ref			
Smoking status									
Current smoker	1.098	0.891	1.353	0.379		−0.013	−0.029	0.003	0.118
Former smoker	1.066	0.862	1.319	0.554		0.000	−0.017	0.016	0.958
Nothing	1.000					ref			
Alcohol consumption									
Drinker	0.678	0.557	0.824	<0.0001		−0.006	−0.021	0.010	0.484
Former drinker	1.089	0.869	1.364	0.460		0.000	−0.018	0.018	0.992
Nothing	1.000					ref			
Stress status									
Hardly feel	0.297	0.223	0.395	<0.0001		0.128	0.111	0.146	<0.0001
Moderate	0.514	0.441	0.599	<0.0001		0.055	0.043	0.066	<0.0001
Almost feel a lot	1.000					ref			
Disability grade									
1–3	1.429	1.206	1.695	<0.0001		−0.008	−0.021	0.006	0.273
4–6	1.000					ref			
Disability type									
Physical disability	1.312	1.125	1.530	0.001		0.009	−0.003	0.021	0.132
Other	1.000					ref			
Year									
2016	1.180	0.997	1.396	0.054		−0.017	−0.030	−0.004	0.012
2017	0.923	0.776	1.098	0.366		0.004	−0.010	0.017	0.590
2018	1.000					ref			

## Discussion

The purpose of this study was to investigate the effects of job satisfaction on the SRH and happiness of PWD working in South Korea. We conducted a longitudinal analysis using data obtained between 2016 and 2018, the 2nd wave of the Panel Survey of Employment for the Disabled.

Our results indicated that as job satisfaction evaluated according to factors such as salary, job stability, the nature of job, etc., decreased, the negative SRH of PWD increased, and levels of happiness that affected their mental health deteriorated. This tendency was consistent with general job satisfaction measured using a single item, resulting in negative SRH and diminished happiness.

The finding that the job satisfaction of PWD is related to SRH as well as happiness, affecting their mental health is consistent with the results of previous studies with non-PWD (24–26). Research investigating the relationship between job satisfaction and individual happiness and health levels is actively being conducted (34, 35). According to a study conducted in Denmark, analyzing the relationship between job satisfaction and SRH (SRH) among 3,727 participants, it was found that the likelihood of reporting negative SRH was 1.78 times higher in individuals who experienced employment insecurity. Additionally, among groups with limited employment opportunities, the SRH of women was found to worsen by 2.13 times (36). Furthermore, according to previous research analyzing the quality of life and happiness among PWD in Korea (25), it was found that for PWD, an increase of one unit in job satisfaction and well-being was associated with a 0.52-point increase in happiness and a 0.25-point increase in quality of life.

As a result of these factors, Korea has established legal safeguards such as mandatory employment of disabled individuals. However, the scale and enforceability of these measures are not significant, and there is a prevailing negative attitude toward hiring PWD within the corporate culture. Consequently, the job satisfaction of PWD (including employment stability, salary, and work environment) is classified as a key influencing factor on their personal happiness (37). Specifically, the mandatory employment system for disabled individuals in Korea requires companies to employ 2–3% of their total workforce as disabled employees. However, the actual disability employment rate is 1.92%, falling short of the legally mandated allocation rate (37). According to a report by the Korea Employment Agency for the Disabled, the societal atmosphere creates significant challenges for PWD to engage in work activities. Even for those who are able to participate in work, they often find themselves in unsatisfactory work environments, where the focus is solely on performing work activities without ensuring a conducive working environment (38). In contrast, Japan, Germany, and the United States have shown higher disability employment rates compared to Korea, with rates of 2.3, 4.6, and 31.4%, respectively. Moreover, in terms of disability-related welfare expenditure as a percentage of GDP, Korea’s expenditure stands at 0.3%, while Japan (0.6%), Germany (1.3%), and the United States (1.0%) demonstrate a more established social safety net, indicating the need for legal and institutional development targeting disabled individuals in Korea (38).

Thus, job satisfaction may play an important role in increasing positive SRH and happiness, affecting mental health, even for PWD. The relationship among job satisfaction, SRH, and happiness or well-being has been largely investigated for non-PWD and few studies have examined this same multivariate relationship for PWD. Thus, the results of this study, indicating that job satisfaction of PWD is closely related to physical and mental health, are important.

This study has some limitations. First, we were not able to identify a complete causal relationship, although we calculated the GEE model using the panel survey data of employment for PWD. To improve the quality of life of PWD, the possible existence of a solid causal relationship should be investigated in future studies. In a similar vein, it will be necessary to examine which factors of job satisfaction are stronger predictors of good quality of life. If greater job satisfaction of PWD improves SRH and happiness, it will be important to work to increase it. There is no concrete definition of job satisfaction as it is a complicated and multidimensional construct to which many factors are linked (39). Therefore, it is difficult to determine which is the strongest factor related to job satisfaction and help PWD to feel more satisfied with their jobs.

In fact, job security, one of the factors used to assess job satisfaction in this study, has shown mixed results for non-PWD. While Hellgren and Sverke (2003) (40) found that job insecurity had a negative effect on the mental but not physical health of workers, Burgard, Brand, and House (2009) (41) found that job insecurity had a stronger association with poorer SRH but not with mental health. Thus, it might be necessary to examine which factors of job satisfaction are associated with better SRH and happiness for PWD. Additionally, demographic and health risk variables of PWD were measured by a self-report survey and thus, the data used in this study can potentially be affected by misrepresentation. Furthermore, it is necessary to measure SRH and happiness more accurately from various perspectives because the variables used in this study were assessed based on a single item in the panel data. in future research, there is a need to utilize objective measures that reflect various perspectives on SRH or mental health (such as PHQ-9 for depression, CES-D for cognitive function). Finally, we attempted to adjust a range of variables using the second wave panel survey of employment for PWD, but most likely we failed to consider all variables which potentially affect health status. There also might have been undetected confounding variables. Lack of such information may have resulted in underestimations or miscalculations in some results of this study.

Although this study may have some Strength, we did find a significant association between job satisfaction of PWD in South Korea and their SRH and happiness, respectively. The results of this study might serve as a foundation for improving the quality of life of PWD in South Korea.

## Conclusion

The results of a longitudinal analysis from the panel data showed that the job satisfaction of PWD negatively associated with SRH as well as happiness in mental health. The findings of this study, consistent with previous research (10, 25, 34, 35), demonstrate that job satisfaction among PWD influences mental health factors, particularly subjective mental health and happiness, which are part of the measurement of quality of life.

To the best of our knowledge, this study is the first of its kind conducted in low-coverage Asian countries targeting PWD. Consequently, it enables us to understand the impact of job satisfaction among PWD in Asia on their SRH and happiness. Furthermore, while previous studies have demonstrated that improving job satisfaction among PWD enhances their quality of life, this study specifically identifies the positive impact of job satisfaction on SRH and happiness.

This study, therefore, suggests that increasing job satisfaction of PWD possibly leads to decreased negative SRH and to increased happiness, resulting in better quality of life. Furthermore, it suggests the establishment of systemic, policy-oriented measures to enhance the employment opportunities for disabled individuals and create an inclusive working environment that aligns with their respective job responsibilities by cooperating between labor authorities and public health authorities. Through this, it is anticipated that the current low employment rate of disabled individuals in Korea can gradually increase, leading to an improvement in happiness-related indicators and economic indicators.

## Data availability statement

Publicly accessible data were analyzed in this study. This data can be found here: https://edi.kead.or.kr/ENG_Contents.do?cmd=_004A&mid=108.

## Ethics statement

Ethical review and approval was not required for the study on human participants in accordance with the local legislation and institutional requirements. Written informed consent from the patients/participants OR patients/participants legal guardian/next of kin was not required to participate in this study in accordance with the national legislation and the institutional requirements.

## Author contributions

YLL designed this study, performed statistical analysis, and drafted the manuscript. JMY contributed to drafted the manuscript. J-HK conceived, designed, and directed the overall study. All authors contributed to the article and approved the submitted version.

## Conflict of interest

The authors declare that the research was conducted in the absence of any commercial or financial relationships that could be construed as a potential conflict of interest.

## Publisher’s note

All claims expressed in this article are solely those of the authors and do not necessarily represent those of their affiliated organizations, or those of the publisher, the editors and the reviewers. Any product that may be evaluated in this article, or claim that may be made by its manufacturer, is not guaranteed or endorsed by the publisher.

## Abbreviations

PSED: Panel Survey of Employment for the Disabled
GEE: Generalized Estimating Equation
